# The Role of Animal Models for Research on Severe Malaria

**DOI:** 10.1371/journal.ppat.1002401

**Published:** 2012-02-02

**Authors:** Alister G. Craig, Georges E. Grau, Chris Janse, James W. Kazura, Dan Milner, John W. Barnwell, Gareth Turner, Jean Langhorne, on behalf of the participants of the Hinxton Retreat meeting on “Animal Models for Research on Severe Malaria”

**Affiliations:** 1 Liverpool School of Tropical Medicine, Liverpool, United Kingdom; 2 Vascular Immunology Unit, Department of Pathology, Bosch Institute, Sydney Medical School, The University of Sydney, Sydney, New South Wales, Australia, and La Jolla Infectious Disease Institute, San Diego, California, United States of America; 3 Leiden Malaria Research Group, Parasitology, Leiden University Medical Centre, Leiden, The Netherlands; 4 Center for Global Health and Diseases, Case Western Reserve University, Cleveland, Ohio, United States of America; 5 Blantyre Malaria Project, College of Medicine, University of Malawi, Blantyre, Malawi, and The Brigham and Women's Hospital, Boston, Massachusetts, United States of America; 6 Centers for Disease Control and Prevention, Atlanta, Georgia, United States of America; 7 Centre for Tropical Medicine, Nuffield Department of Medicine, Oxford University, United Kingdom, and Mahidol-Oxford Tropical Medicine Research Unit (MORU), Faculty of Tropical Medicine, Mahidol University, Bangkok, Thailand; 8 Division of Parasitology, MRC National Institute for Medical Research, London, United Kingdom; The Fox Chase Cancer Center, United States of America

In light of the recent controversies over the role of animal models for research into the development of new treatments for severe malaria, particularly cerebral disease, a group of scientists came together to discuss the relative merits of a range of animal models and their overlap with the complex clinical syndromes of human disease. While it was not possible to fully resolve differences over the utility of the *Plasmodium berghei* ANKA model of experimental cerebral malaria, the meeting did bring the two research communities closer together to identify further work to provide information needed to validate the model and revitalise the development of other animal models displaying features of human pathology. The driving force behind this was the desire to ensure better translation of experimental findings into effective treatments for severe malaria.

## Introduction

In 2010 there were a number of publications discussing the relevance of rodent models for the study of human cerebral malaria (HCM). These were prompted by an initial discussion paper from Professor Nick White and colleagues (“The murine cerebral malaria phenomenon” [1]) in which the suggestion was made that the *Plasmodium berghei* ANKA (PbA) mouse model of experimental CM (ECM) did not replicate the pathophysiology of cerebral disease in humans, and which was followed closely by a set of papers supporting the ECM approach [2]–[5]. This has resulted in some polarisation of the research community on this topic and an occasionally uncritical application of some of the perceived conclusions of this initial paper to other areas of malaria research using rodent animal models. This Wellcome Trust–supported workshop brought together experts (Table 1) from both sides of the debate and those with expertise related to non-human primate (NHP) malaria to discuss the clinical features of severe malaria in humans and how various animal models might best be used to advance our knowledge of this important disease and the development of therapeutic measures.

**Table 1:** Participants at the Meeting.

Paul Hagan – Scottish Funding Council, UK
John Barnwell – Centers for Disease Control and Prevention, USA
Jim Brewer – University of Glasgow, UK
Alister Craig – Liverpool School of Tropical Medicine, UK
Brian de Souza – UCL Medical School and London School of Hygiene and Tropical Medicine, UK
Patrick Duffy – National Institutes of Health, USA
Chris Engwerda – Queensland Institute of Medical Research, Australia
Mary Galinski – Emory University, USA
Georges Grau – University of Sydney, Australia, and La Jolla Infectious Disease Institute, San Diego, USA
May Ho – University of Calgary, Canada
Lars Hviid – University of Copenhagen, Denmark
Chris Janse – Leiden University Medical Center, The Netherlands
Jim Kazura – Case Western Reserve University, USA
Clemens Kocken – BPRC, The Netherlands
Jean Langhorne – MRC National Institute of Medical Research, UK
Dan Milner – Blantyre Malaria Project, Malawi and The Brigham and Women's Hospital, Boston, USA
Chris Newbold – The Weatherall Institute of Molecular Medicine, Oxford, UK
Laurent Renia – A*Star, Singapore Immunology Network, Singapore
Eleanor Riley – London School of Hygiene and Tropical Medicine, UK
Gareth Turner – Mahidol-Oxford Tropical Medicine Research Unit, Mahidol University, Thailand
Nick White – Mahidol-Oxford Tropical Medicine Research Unit, Mahidol University, Thailand
Sanjeev Krishna – St. George's, University of London, UK

## What Are the Clinical and Pathological Features of Severe Malaria?

An important aspect of falciparum malaria that has not been always fully appreciated is the heterogeneity of disease patterns in humans. Frequently the terms “severe” and “cerebral” when used in conjunction with the term “malaria” have been thought to be interchangeable, but severe malaria is made up of a range of clinical syndromes, several of which do not involve coma. Severe malaria shows both clinical heterogeneity between patients and the patterns of pathophysiology observed between adults and children. Therefore it is important when dealing with studies on severe malaria that a reliable and consistent definition of disease is used. Detailed clinical criteria for the diagnosis of severe malaria have been defined by the World Health Organization [6].

Most studies on severe malaria have focused on infection by the malaria parasite *Plasmodium falciparum*. Adults with severe malaria show signs of profound metabolic derangement and cerebral malaria, and may have placental malaria and (unlike children) multi-organ failure, including kidney and liver dysfunction. For children, severe disease includes metabolic derangement, cerebral malaria, and severe malarial anaemia. Frank pulmonary oedema is less common in children. Severe malaria (particularly lung involvement, thrombocytopenia, or anaemia, but without coma), although less frequent, can be caused by *Plasmodium vivax* [7], [8]. *Plasmodium knowlesi* cases also frequently develop severe malaria, with limited clinical data and autopsy findings from a single case report appearing to show similarities to *P. falciparum* pathology but without coma [9].

A principal feature of HCM from nearly all post-mortem studies is the packing of sequestered infected erythrocytes (infected red blood cells [iRBC]) in the brain microvasculature [10], [11], associated with microvascular obstruction [12]. This may also be associated with microhaemorrhages, although these are also seen in non-cerebral malaria cases and other diseases, and thus are not specific. The proportion of brain vessels containing sequestered iRBC can range from 30% to 100%, indicating heterogeneity in the recruitment of iRBC to receptors on the endothelial cells lining these vessels [13], [14]. The post-mortem studies conducted in Malawi suggested that clinical diagnosis of fatal HCM in an endemic area proved false in 25% of cases on subsequent autopsy, where the cause of death was due to other diseases [15]. The need for more precise clinical and pathological definitions of severe cerebral and non-cerebral malaria is therefore important. Direct visualisation of microcirculatory obstruction (such as in the retinal circulation) and associated signs has helped to provide better specificity in making a diagnosis [16]–[20].

Mortality in paediatric HCM occurs very rapidly, with 50% of deaths being recorded within 24 hours of admission and nearly all fatalities by 48 hours. In adults a similar level of early mortality is observed, with about 50% of deaths occurring in the first 48 hours post admission, but subsequent deaths can take place later due to other complications such as renal failure or incidental pneumonia. Patients with HCM tend to have higher parasitaemias, and some studies have also suggested a larger parasite biomass (as estimated by plasma PfHRP2 levels [21]). However, infections with hyper-parasitaemia without manifestations of HCM also occur. A key point in interpreting these findings is the location of the tissue burden of sequestered parasites in severe disease, and the immune mechanisms, that attenuate disease.

Information on the histological pattern of severe malaria is still very restricted, particularly from childhood deaths, in part due to the difficulty in accessing relevant human material (see below). Given the multiple aetiologies of severe disease, this type of information will be critical in helping us to understand the relative contribution of different pathologies to the clinical signs observed in severe malaria. A consistent pathological finding in HCM at autopsy, unique to this disease, is the presence of sequestered iRBC that pack brain blood vessels. Indeed, patients who die soon after admission to a health care facility may have brain microvascular sequestration as the only significant pathological feature of CM. However, observation and correlation of neuropathological features with pre-mortem clinical diagnosis can be affected by multiple factors such as time to death, treatment, and other systemic complications of severe malarial infection, or may be compromised by misdiagnosis. In this regard there are some differences between adults and children, for example, in the degree of brain swelling and oedema observed using imaging techniques, and the accumulation of leukocytes and platelets in cerebral microvessels, with significant levels seen in some cases of paediatric HCM but very few observed in adults, despite clear evidence (for instance, from circulating cytokine levels) of a vigorous pro-inflammatory host response. Astroglial activation and blood–brain barrier changes are also commonly observed in HCM, so inflammatory responses elicited within the brain parenchyma may be more subtle than merely accumulation of monocytes or other leukocytes [22], [23]. Data are also available for iRBC sequestration and host responses in non-brain tissues that represent other sites for maturation of *P. falciparum* within erythrocytes [10].

Adhesion-related pathology in falciparum malaria is evident in placental infections in pregnant women. Here, placental sequestration of iRBC and the onset of fetal impairment and disease in women protected by immunity is linked to the appearance of a set of parasite variants expressing a class of PfEMP1 proteins known as VAR2CSA [24] with atypical structure and domain composition [25], [26]. A key feature of these parasite variants is their ability to bind to chondroitin sulphate A (CSA) in the placenta [27], where they are thought to induce fetal disease through as yet unknown inflammatory pathways [28].

## Important Issues in Research on Severe Malarial Disease and the Use of Animal Models

Two key questions, “what is the pathophysiology of severe disease?” and “how do people living in malaria endemic regions become protected from severe disease?”, cannot be answered fully by observations of infected humans. While studies on the clinical definition and histopathology of HCM and placental malaria have provided information regarding correlates of pathogenesis, the exact mechanisms whereby malaria parasites cause severe disease and how immunity protects against disease despite infection remain unknown. Parasites with complex life cycles such as *Plasmodium* species have evolved multiple and redundant strategies to deflect and evade the host immune response, so it is unlikely that protection will be correlated with the ability to recognise and neutralise a single antigen [29]. In the case of *P. falciparum*, anti-disease immunity develops relatively quickly, perhaps after only a few infections, but this immunity is incomplete, still allowing lower density blood stage infection that is accompanied by either mild or no symptoms [30]. Immunity is also linked to levels of exposure so that children appear to acquire anti-disease immunity at a very early age in areas of high transmission (and have a distinct pattern of severe disease, dominated more by severe malarial anaemia rather than HCM) [31]. By contrast, malaria naive adults and older children encountering the parasite for the first time produce a different defence response. Linked to immunity is the question of the role of inflammation in the pathogenesis of severe disease. Investigations of this issue require not only longitudinal clinical studies, but also the analysis of multiple host responses. Thus, it is no surprise we do not fully understand the mechanism of protection either from severe disease or infection itself.

Mechanistic studies in animal models may provide information on the processes of, and protection from, severe malarial disease, and there are good examples where they have provided insights into features and characteristics of human malaria infection. Although it is unlikely that any single model will reproduce the complexity and spectrum of disease and immunity observed in human malaria infections, there are parallels between some human and animal presentations of malaria infection (and disease). However, the choice of the host/parasite combination is important (see Table S1).

### Validity of the Rodent *P. berghei* ANKA Model (PbA Mouse Model) for Human Cerebral Malaria?

In some ways, this is one of the most challenging questions for the malaria research community and accordingly has resulted in a degree of polarisation of viewpoints. The correct choice of a combination of experimental animal host and a malaria parasite able to appropriately mimic the disease pattern seen in humans is essential. However, it is also very clear that animal models allow for more detailed examination of multiple and specific patholphysiologic processes caused by malaria infection that is not possible for clinical studies of human malaria through the level and scope of experimental observation and intervention that can be applied to animals. In discussing the role of the PbA mouse model of HCM specifically, the issue that recurred throughout the meeting concerned the degree of sequestration of iRBC in the brains and other organs of *P. berghei* ANKA–infected mice. Several groups provided data that supported the accumulation of iRBC in a number of organs, sometimes including the brain, and that higher parasite accumulation was seen in mice susceptible to ECM. However, the key piece of information missing was direct histological evidence of substantial parasitised erythrocyte sequestration packing brain microvessels in a similar pattern to that observed with HCM.

The consensus of most participants was that *P. berghei* ECM, in contrast to HCM, is associated with marked accumulation of leukocytes, but not with prominent sequestration of cytoadherent mature trophozoite/schizont iRBC in brain vessels [32]. However, a number of participants emphasised that recent data have provided evidence of increased iRBC accumulation during ECM in multiple organs, including the brain [4]. It was felt that more research is needed to define this phenotype of iRBC accumulation during ECM.

ECM is associated with increased accumulation of immune cells in the brain (particularly monocyte/macrophages and T cells). In particular, recruitment of CD8+ T cells to the brain late in disease pathogenesis, and production of the cytolytic molecules granzyme B and perforin, is critical for ECM development [33]. In general, it was agreed that in the absence of prominent parasitised erythrocyte sequestration in the brain it is not appropriate to use the PbA mouse model for testing interventions that may reverse iRBC adhesion to relieve microvascular obstruction (a hallmark of HCM). However, since in both humans and rodents vascular obstruction, either by iRBC, leukocytes, or by both, appears to be associated with features of CM, the PbA mouse model may be useful to analyse the relationship between reversal of vessel blockage and inflammation in CM. The types of endothelial cell microenvironment changes induced by cytoadherence and inflammation are not the same [34], although they share several processes, and as HCM encompasses more than one pathologic entity it is possible that disease may be caused by different vascular conditions. Thus, there is room for investigating these different mechanisms in relation to human disease. For HCM, both post-mortem and in vitro evidence show that cytoadherence of iRBC to brain endothelium has specific functional effects [35]–[37].

ECM is clearly an inflammatory syndrome with local vascular endothelial activation with inflammatory cytokines playing an essential role, which has obvious differences and some similarities to the clinical and pathological features of HCM, for example, the signs of vascular inflammation/damage seen in some cases of paediatric HCM [15]. Some studies have suggested associations between high levels of cytokines and severe malaria [38], [39], but these have been challenged recently by work showing that high levels of pro-inflammatory cytokines such as tumor necrosis factor (TNF) are poor indicators of HCM in African children and that markers of localised endothelial cell inflammation (e.g., Angiopoietin 1/Angiopoietin 2 ratio) are more strongly associated [40], [41]. It is possible that inflammatory processes play an essential role in a subset of clinical patients. Therefore, (mechanistic) studies of ECM in PbA mouse models may provide insights into inflammatory processes and/or immune responses and their role in HCM pathology. An alternate view, strongly articulated at the meeting, was that ECM in the PbA mouse model was so very different to HCM that the pathological and therapeutic interpretations derived from it were largely irrelevant for the understanding and treatment of HCM. It was suggested that future experimental studies in the mouse and other animal models (i.e., NHPs) would be better directed towards pathological processes that could be shown to be similar in human infection and the animal models. Other features of ECM, such as PbA-iRBC adhesion to CD36, may have relevance to HCM. Although CD36 is expressed at very low levels on cerebral endothelial cells in the human and mouse, it is abundantly expressed on platelets, macrophages (cells that accumulate in the brain in ECM), on endothelium in other vascular compartments, and is also present on microparticles. The role of platelets and microparticles in human malaria has become a major topic of research [42], [43] and may become more relevant to identifying potential interventions for HCM.

One of the benefits of the discussion about ECM studies at the meeting was a clarification of how inhibitor or intervention studies of adjuvant treatments in mice should be interpreted. In most studies, the inhibitors/drugs are given before the development of neurological symptoms, thereby providing information on disease processes in ECM but not necessarily on the identification of viable therapies. The validity of carrying out human clinical trials, often in underpowered studies, purely on the basis of this type of rodent malaria data is questionable. On the other hand, human trials carried out on the basis of pathophysiology studies in humans (e.g., anti-TNF [44]; anti-convulsion therapy [45]; fluid management [46]) have not been particularly good at reducing morbidity or mortality either, so the lack of progress in this area may reflect our lack of knowledge and the complexity of HCM.

Another benefit of the reappraisal of ECM at the workshop was the recognition of the diversity of phenotypes available with rodent model systems through the use of different protocols and *P. berghei* isolates/strains. For example, the level of ECM induced by *P. berghei* infections (i.e., the percentage of mice showing ECM pathology) is influenced by (small) differences between isolates/strains and cloned laboratory lines (i.e., NK65, K173, and ANKA [47]), host diet [48]–[50], starting dose of infection, etc. Parasites of strains such as NK65 and (cloned) lines of other strains that have a stronger preference for invading reticulocytes appear to have a lower capacity to induce ECM. Therefore, standardisation of protocols for maintaining and generating the genetic characterisation of parent parasite lines is essential, as is the analysis of multiple infection parameters in studies that analyse interventions aimed at reducing ECM.

### What Other Rodent Models of Human Cerebral Malaria Are Available or Possible?

With questions raised about the relevance of the PbA mouse model for HCM, a consideration of alternative rodent models was undertaken. Improvements based on genetic manipulations to the mouse model were discussed, such as a humanised rodent system. Schizonts of *P. berghei* sequester and express parasite ligands on the surface membrane of iRBC [51]. The characterisation and the genetic modification of *P. berghei* ligands involved in binding to host receptors might offer novel possibilities in the development of small-animal models for analysing the sequestration properties of *P. falciparum* ligands that have hitherto only been examined in vitro. This could be performed by, for example, substituting *P. berghei* ligands with PfEMP-1 proteins or domains. Using in vivo imaging in conjunction with such “falciparumised” *P. berghei* parasites in mice expressing human receptors (e.g., human ICAM-1) may create an in vivo screening system for testing inhibitors that block *P. falciparum* cytoadherence. While this “double transgenic” model is not available yet, human graft systems have been investigated using SCID mice, as transient models allowing perfusion of P. *falciparum* iRBC in human dermal tissue in a format where detailed microscopy is facilitated [52], and in investigating the role of Fc receptors in malaria [53]. The graft model allows detailed examination of the interaction between parasites and the host in a three-dimensional tissue, rather than the two-dimensional ex-vivo models commonly used. Complex humanised models for longer-term experiments on *P. falciparum* infections in NOD/SCID mice have also been described [54]. Results suggest that these do not show cytoadherence [55] or ECM, and so they cannot be used for HCM research. Uptake of graft models has been extremely limited and further work is needed to validate their potential use and to develop the protocols to extend the range of tissues available.

### Non-Human Primate Models for HCM or Severe Malarial Disease

Given the greater genetic relatedness of primate hosts and primate parasites, the importance of NHP and malaria parasite combinations as appropriate systems for researchers to study ECM was also discussed at the workshop. Further, it was noted that a degree of reluctance to use NHP models in malaria research has led to a reduction in the resources needed to support such research. If NHP systems are capable of providing models of HCM and other types of severe disease in human malaria, then significant investment will be required to grow and sustain this research community, train young researchers, and provide the resources for wider participation of clinical and laboratory oriented investigators.

Several host/parasite combinations were discussed (Table 1): *Plasmodium coatneyi* is a simian parasite that in Japanese (*Macaca fuscata*) and rhesus (*Macaca mulatta*) macaques exhibits phenotypic characters that mimic *P. falciparum* iRBC sequestration, rosetting, and clinically severe disease [56]–[60]; similarly, *Plasmodium fragile* iRBC profoundly sequester by cytoadhesion in the vasculature of various tissues and organs and produce severe disease in rhesus monkeys [61], [62]. These two malaria parasite species, like *P. knowlesi*, but not *P. berghei* and other rodent malarias, have *var*-like gene families that exhibit antigenic variation and adhesion properties [63]–[65]. In contrast to the PbA mouse model, the presence of cerebral microvascular iRBC sequestration through cytoadhesion in cerebral blood vessels is proven for these two macaque monkeys, and they therefore offer an alternative for examining the association of parasite-specific adhesive events in the brain with disease and underlying pathogenic mechanism(s). These models also offer the potential to use more sophisticated approaches for neuroimaging, such as functional MRI [66], which has been applied to HCM [67], [68] and in initial studies of *P. coatneyi* malaria [69].

*P. knowlesi* infections in *M. mulatta* produce “severe disease” that is almost always lethal to this host. Mature *P. knowlesi* iRBC in rhesus monkeys partially sequester in a variety of tissue sites via the variant-antigen products of the large *var*-like SICAvar multi-gene family [64], [65]. Although it is clear that severe disease in the *P. knowlesi* rhesus macaque model can mimic some clinical syndromes of *P. falciparum* severe disease in humans, it is questionable whether it is a useful model for HCM, as neither adhesion of iRBC to cerebral vessels or clinical signs of HCM have been noted. Although it is unclear if *P. knowlesi* in rhesus monkeys is a satisfactory model for HCM, from initial reports it is certain to be a model for human severe disease caused by this parasite [9], [70], [71]. *P. knowlesi* in the olive baboon (*Paplio anubis*) [72] has been suggested as an HCM model because of apparent cerebral iRBC sequestration and neurological signs in terminal infections, but this needs further investigation. *P. falciparum* will also infect and cause severe disease (anaemia, acute nephritis, cardiopathy, metabolic collapse) and death in neotropical monkeys such as various species of *Aotus* (owl monkeys).

There are, however, limitations to this primate model since symptoms of CM and cerebral vascular sequestration have not been noted in New World monkeys infected with *P. falciparum*.

### What Models Are Available for Other Aspects of Malaria Research?

At the meeting, brief mention was made of models for placental malaria, acute respiratory distress (ARDS), anaemia, and immunity. The lack of the *var* multi-gene family in rodent and some primate malaria species (i.e., *Plasmodium cynomolgi*, which is a model for *P. vivax*) means that the roles of the encoded variant antigens in placental sequestration cannot be investigated. However, NHP models such as *P. coatneyi* in rhesus monkeys [73] or *P. falciparum* in *Aotus* monkeys are also available; and *P. coatneyi* expresses the comparable variant antigen multi-gene family (J. Barnwell and M. Galinski, unpublished data). Furthermore, analysis of the effects of inflammatory responses and parasite accumulation/sequestration in the placenta and foetus may provide insights into the role of pro-inflammatory cytokines in malarial placental pathology [74]–[77]. Major limitations of many mouse models are the lack of a natural chronic infection, with the exception of *Plasmodium chabaudi*, and the ability of some host/parasite systems to develop almost complete immunity after a single infection. Investigation of the effects of malaria on multiple pregnancies is therefore not possible in most rodent models except in drug-induced chronic *P. berghei* infection in mice, which is one of the best described models for rodent placental malaria. A “humanised” PbA mouse model may also be developed for analysing sequestration properties during pregnancy. Transgenic *P. berghei* parasites may be generated that express Pf VAR2CSA or CSA binding domains on the surface of iRBC. Such parasites in combination with a “humanised placental malaria mouse model” may offer a screening system for in vivo testing inhibitors (small molecule inhibitors, antibodies) that block *P. falciparum* sequestration.

Several recent studies indicate that *P. berghei* in mice can be models to investigate malaria-associated lung pathology [78]–[81]. For these models it was felt that, as with CM, detailed comparisons between lung pathology in the rodent models and lung pathology in humans are required to validate their use for developing therapies/interventions.

Models for anaemia were also considered to be important to support human clinical studies. *P. chabaudi* [82] and *P. yoelii* 17xNL in mice and *P. berghei* in Brown Norway rats (C. Janse and B. Franke-Fayard, unpublished data) were briefly mentioned as possible models for malarial anaemia. However, the lack of a real chronic infection and the rapid acquisition of sterilising immunity preclude the use of many rodent models to study anaemia in chronic infection, which would be more reflective of severe malaria in children. However, mechanisms of disruption of bone marrow haematopoeisis [83] and dyserythropoeisis in acute infection can be readily investigated.

Based on mainly unpublished reports, several NHP models offer opportunities for studying severe anaemia in both acute and chronic infection in regards to clarifying the mechanisms and interventions for clearance dynamics of uninfected RBC, bone marrow differentiation, and dyserythropoesis in severe malarial anaemia. Simian malarias such as *P. coatneyi* in acute infections produce profound anaemia that is life-threatening due to clearance of uninfected erythrocytes and a dyserythropoesis that does not allow a compensatory reticulocytosis (A. Moreno and M. Galinski, unpublished data), as well as severe anaemia later on during chronic severely recrudescing infections that can last for months or over a year (W. Collins, unpublished data). *P. cynomolgi*, like *P. vivax*, also produces a profound anaemia during acute infections (A. Moreno and M. Galinski, unpublished data). Severe lethal anaemia also develops in semi-immune neotropical primates with *P. falciparum* infections going into chronic infection and upon reinfection or challenge of animals induced into a semi-immune state (J. Barnwell and W. Collins, unpublished data). *P. vivax* infections in neotropical monkeys also develop severe anaemia during acute and chronic infections [84], [85].

It was generally agreed that rodent models can provide relevant information on mechanisms of host defence and immunity to all stages of the infection. A recent example of this is the demonstration of suppression of liver stage malaria parasite development in the presence of a blood stage infection [86]. *P. chabaudi* and P. *yoelii* in mice and *P. berghei* in Brown Norway rats show self-resolving infections with low peak parasitaemias and anaemia. It is generally assumed that immunity in humans and NHPs develops slowly over many months and incompletely, in contrast to rodents where immunity develops rapidly and is frequently sterilising. Only *P. chabaudi* in mice [87] and *P. berghei* in rats exhibit low density chronic infections for prolonged periods (2–3 months), whereas in humans and NHPs single infections may last years and decades. One feature in common to human, NHP, and rodent infections is that protective immune responses are generally species-, genotype-, and variant-specific, although in the case of variant-specific immunity some of the antigens that are products of multi-gene families (e.g., PfEMP1 and PIR) exhibit evolutionarily distinct sequences and characteristics in the species and strains where they exist. Humans are generally exposed to different strains, genotypes, and variants of the parasite, whereas in most experimental model studies the same cloned parasite line or a single strain/isolate is used for repeated infections. It was emphasised that when humans are similarly re-exposed to the same strain/genotype, acquisition of immunity is generally similar to that of mice. A recommendation therefore was that studies on immunity to re-infection and the effectiveness of immunological memory should be carried out in mice or monkeys using different strains of the same parasite species. More immunological studies in mouse/rat peripheral blood, for comparison with humans, would also be valuable. Another important point of experimental model studies was that direct injection of infected erythrocytes to initiate infections was normal practice in the study of immune mechanisms and immunopathology. However, this does not reproduce the sequence of the natural infection where infection starts with a mosquito bite and inoculation of sporozoites. It was discussed here that blood stage infections of some *Plasmodium* species may look very different and be considerably less virulent when initiated via the natural route compared with injection of iRBC. This should be taken into account when translating findings from animal models to humans.

At the meeting the use of animal models for development and testing of vaccines and drugs was only briefly mentioned. Discussion of this area was outside the scope of the meeting, but it was acknowledged that animal models have played a major role in the development of anti-malarial therapies, including those targeting different stages in the parasite life cycle [2].

## Conclusions and Recommendations

**There was universal support for stronger interactions between groups working on human and animal studies, both to improve current models and to promote more realistic translation of laboratory findings into clinical studies.**

The importance of animal models in malaria research was recognised by all participants. However, it was not possible to reach a consensus on the role of the PbA mouse model for studies on HCM. The basis of the disagreement centred on the relative importance of iRBC adhesion to brain endothelial cells in causing HCM pathology. There is evidence to support the presence of PbA iRBC in cerebral vessels in ECM, but this accumulation represents a minor (if any) concentration of iRBC compared to the peripheral parasitaemia, unlike the levels of adherent iRBC seen with *P. falciparum*. In the absence in the PbA mouse ECM model of this hallmark of human CM, and based on observations of pathologic changes to endothelial cells on interaction with iRBC, it was the view of some of the participants that the mouse/PbA system was not a useful model of HCM. However, it was pointed out that we do not know that *P. falciparum* cytoadherence causes HCM and its role in pathology may have been exaggerated. At the same time, comparisons of ECM and HCM have revealed many similarities (although the extent of these was also contentious), and therefore the PbA mouse model can provide a platform for detailed mechanistic experiments in this field. Thus, the overriding conclusion of this meeting was that there are presently genuine differences of opinion on this topic, but this was coupled with the genuine willingness indicated by all parties at the meeting to try overcoming these differences by collaborating. Specifically, these centered around two major areas: 1) A more careful use of the term “drug” when applied to interventions used in ECM, particularly when these were applied prior to the physiological signs of cerebral disease in mice, although the validity of the use of inhibitors to understand pathological processes was appreciated. 2) A bi-directional exchange of resources and knowledge to provide better information about the complex pattern of human disease to scientists using animal models and better access for this latter community to human tissues in order to validate findings obtained from model systems. The major issues raised at the meeting are summarised in Box 1.

**Box 1. Major Issues from the Meeting**

- The mouse and human malaria research communities need to work together more closely to support better translation of laboratory studies into clinical leads.
- It was not possible to resolve the disagreements over the use of the *P. berghei* ANKA mouse model for human cerebral malaria, particularly in the area of cytoadherence-associated pathology. It was agreed that researchers using the *P. berghei* model should be cautious in their interpretation of inhibitor studies, particularly where the intervention is given prior to symptoms.
- The spectrum of malarial disease in humans is broad and we do not fully understand the pathological mechanisms.
- Development and standardisation of animal models, including renewed investment in non-human primate systems, is a priority.

**The main recommendations from the meeting were:**

**(i) Coordinated pathological studies across species**

As with many disciplines, one of the major confounders of research is the lack of standardisation. This is particularly clear with the mouse/PbA model, where differences between parasite lines and environmental factors, such as host diet, can influence features of pathology. Thus, there was a general consensus that tissue specimens from animal models of CM produced in different laboratories need to be analysed using uniform methods of histology and quantification. While it was questioned whether the mouse/PbA model is appropriate to derive cytoadhesion-based therapies for CM, it does not preclude this model as a vehicle to investigate possible inflammatory processes in severe disease, including CM. Further research is needed to link animal and human studies to allow a comparison of the pathology of disease across a spectrum of clinical syndromes and thereby to develop the much needed animal models that can drive research into the development of interventions for severe malaria. The mouse model may be useful in studying tissues where inflammation is known to play a significant role in human pathology, such as the lung. But in some clinically important syndromes of human malaria such as malaria-associated acute renal failure in adults, very little data are available on the clinical or pathological correlates in animal models. However, NHP infections in rhesus monkeys involving several simian malaria species can cause acute renal failure.

**(ii) More use of primates and better access to primates**

Strengths of studies on NHPs are that their physiology and acquisition of immunity to infection are more similar to humans than mice, and their size may also permit a wider range of studies. Also, within limits, neotropical (New World) primates can be infected with human *P. falciparum* and *P. vivax* (as well as *P. knowlesi*). Infections with *P. fragile*, *P. coatneyi*, and *P. knowlesi* in certain macaque species are potential models of CM, anaemia, placental malaria, and ARDS and other severe malaria syndromes in humans [88]. NHP models can also facilitate studies of malaria parasite multi-species co-infections and co-morbidities, e.g., affect of worm infections on the severity of malaria, and overlapping syndromes, e.g., metabolic acidosis, anaemia, etc. One drawback is that the currently available NHP models, such as *P. coatneyi* in Japanese or rhesus macaques, exhibit cerebral sequestration but it is not clear how similar they are to HCM.

NHP models of vaccine efficacy (using GMP material) have been reasonably predictive of human clinical trials (albeit mainly negative, as are most clinical trials). Unlike rodent models, they do provide access to some of the life cycle stages that are not available in vitro, including hypnozoites.

NHP studies for malaria research require specialised scientific centres with the regulatory framework, access to NHP animals, and expertise to conduct such research. The group recognised that investment in existing NHP research centres would be a valuable and cost-effective way forward, coupled with a scheme to provide “placements” for scientists from collaborating laboratories. This would ensure an enhanced and vibrant scientific environment for malaria research within the NHP centres and disseminate the potential for NHP research to the community.

**(iii) Clear discrimination between intervention and mechanistic studies**

One of the more fundamental problems of animal models highlighted by recent publications has been the confusion between studies aimed at collecting mechanistic information on disease processes and studies aimed at screening for potential therapies against severe disease, perhaps most graphically demonstrated by the figures of 38 of 42 interventions for ECM working but none of 16 being successful in HCM. The limitations associated with the application of therapies before clinical signs, often used in PbA mouse model studies, need to be recognised. Relevant animal models are highly desirable, as studies of humans with severe malaria are by definition limited to situations where clinical interventions are the priority. Thus, interpretations of the result of therapies preventing CM must be done in the context of anti-malarial drug treatment and other supportive measures.

**(iv) Facilities to support research on severe malaria**

One of the major outcomes of the workshop was the requirement for further development of facilities for research. We need to understand more about human disease and immunity and communicate more effectively with researchers working on animal studies so that we can identify appropriate models for detailed mechanistic studies. Apart from a general need for more work in this area, two specific areas were identified for further development:

**(v) Tissue biobanking**

Our lack of knowledge of the detailed pathology of human disease is part of the problem in identifying relevant animal models. To address this, the creation of a tissue biobank was proposed. This resource would make material from humans with malaria available to the broader research community so that validation of initial observations from well-characterised clinical specimens could be examined from a variety of perspectives using novel technologies. There are clearly ethical and logistic issues associated with this, but the benefit accrued in terms of validating animal studies by having good access to human tissues was deemed to outweigh the effort that would be required to set this up properly. The use of tissue microarrays, where multiple cases can be examined on a single slide, may facilitate this approach.

**(vi) Standardisation of animal models**

It was proposed that a repository of standard parasite lines and protocols relating to the maintenance/passage of parasite lines and their hosts should be created. There should also be an opportunity for members of the research community to share information (particularly unpublished data that is not widely available), including data such as a comparison of parasite sequences.

## Supporting Information

Table S1 Animal host/malaria parasite combinations for the study of human malaria pathogenesis.
(DOC)
