# Supplementary material for: The Role of Animal Models for Research on Severe Malaria
Source: PLoS Pathog. 2012 Feb 2;8(2):e1002401. doi: 10.1371/journal.ppat.1002401 (PMC3271056; doi:10.1371/journal.ppat.1002401)
Supplement: Table S1 — Animal host/malaria parasite combinations for the study of human malaria pathogenesis. (DOC) [file ppat.1002401.s001.doc]

| ***Syndrome/ severe manifestation*** | ***SUGGESTED MODELS*** | | | |
| --- | --- | --- | --- | --- |
|  | ***Rodent*** | | ***Non-human primate*** | |
|  | ***Parasite/host*** | ***Remarks*** | ***Parasite/host*** | ***Remarks*** |
| **Cerebral malaria**:  cytoadherence model with clinical features of *P. falciparum* that reflects:  1) dense iRBC sequestration with adhesion to endothelium by variant Ags and abundant microvascular obstruction  secondary to cytoadherence.  2) typical clinical presentation and sequelae with coma ending in respiratory arrest and death.  Temporal coma model for testing reagents to reverse coma.  Microvascular injury with measurable neurological deficits to test neuroprotective agents | *P. berghei* ANKA in C57BL/6 or CBA mice  *P. berghei* ANKA in C57BL/6 or CBA mice  None  *P. berghei* ANKA in C57BL/6 or CBA mice | CD36-mediated sequestration in lung but no CD36 on human brain endothelium. Sequestration index of iRBC in tissue/iRBC in circulation is low in brain. Need more finely detailed histopathology by LM and EM.  No evidence for iRBC microvascular obstruction. Consistent histopathological evidence for a vigorous inflammatory response in ECM unlike HCM where no inflammation is observed in some cases.  Most interventions tested in the rodent model are beneficial whereas none have proved effective in HCM | *P. coatneyi* in *M. mulatta*  *P. coatneyi* in *M. fuscata*  *P. fragile* in *M. mulatta*  *P. knowlesi* in *P. anubis*  None  None | Cerebral sequestration but inadequate data on frequency and degree of clinically frank CM presentation.  Indications of CM; more data required to validate model of HCM or just severe malaria |
| **Cytoadherence, parasite sequestration and Ag variation**  Host/parasite combination that combines Ag variation and cytoadherence.  Ag switching *in vivo*. | Not known: could be *pir* gene family  *P. chabaudi*/mice | Very low chronic infections, Immune to homologous challenge after one infection | *P. coatneyi* or *P. fragile* in rhesus *M. mulatta*  *P. knowlesi* in *M. mulatta* | No genome sequence and no transfection as yet; however, genome sequencing for *P. coatneyi* is in progress.  *var* gene family (*SICAvar*); genome sequence and transfection available |
| **Other pathology:**  Model of acute renal failure secondary to severe malaria  Model of chronic tubular necrosis secondary to chronic malaria  Hypoglycemia and acidosis with elevated lactate-pyruvate ratios.  Pulmonary edema/ ARDS  Anemia    Pregnancy/placental malaria  in vivo model of placental malaria with VAR2CSA type Ag in animals with preexisting protective immunity | None  None  *P. berghei*/ young rat  *P. berghei* ANKA/DBA/2 mice  *P. berghei* NK65/C57BL/6 mice  *P. berghei*/mice and rats, *P.yoelii*, *P. chabaudi*/mice  *P. chabaudi*/mice  *P. berghei*/mice  *“falciparumerized*” rodent parasite in humanized mice | Hypoglycaemia and acidosis are consistent features of all lethal experimental malarias in mammals and birds as is anaemia  Often related to high parasitaemias- not true in children | P*. falciparum* in owl monkey  *P. knowlesi*  in rhesus monkey  *P. brasilianum* or *P. malariae* in owl or squirrel monkeys; *P. inui* in rhesus monkeys  *P. malariae/A. nancymaae*  *P. coatneyi*/rhesus  *P. falciparum/A. nancymaae*  *P. coatneyi /rhesus* for chronic placental malaria  *P. cynomolgi* or *P. coatneyi* in rhesus for acute infections during pregnancy | Pathology of glomerulonephritis in relapsing human malarias; *P. falciparum* acute renal failure common in adults with acute tubular injury |
| **Immunity:**  *in vivo* model to study efficacy of variant Ags immunization.    Persistent infection  Model in which there is sufficient genotype diversity to examine heterologous challenge.  Models to assess vaccines or drugs | *P. chabaudi*/mice  “*falciparumerized*” rodent parasite in humanized mice.  *P. chabaudi*  *P. chabaudi*  most rodent malaria  species in mice | No *var* genes, but *pir* genes  Sub-patent parasitaemia up to 45-90 days  Immune after single infection to homologous challenge | *P. knowlesi* in rhesus  *P. cynomolgi* in rhesus  *P. inui* in rhesus  *P. knowlesi* in rhesus  *P. cynomolgi* in rhesus  *P. falciparum*/*P. vivax* in *Aotus* and *Saimiri* spp./P. *knowlesi*  *P. cynomolgi/P. vivax* in *Aotus* and *Saimiri* spp.  *P.coatneyi/P. fragile* in rhesus  *P. cynomolgi* in rhesus  *P. knowlesi* expressing Pf or Pv genes in rhesus | *Var* gene family (*SICAvar*) and *kir* gene family  Chronic infection and hypnozoites  Long-term chronic infections  Genome sequence available and many isolates archived  Transgenic parasites not made yet. |

**Table S1.**  Animal host/malaria parasite combinations for study of human malaria pathogenesis
